# Deciphering the Complex Signaling Systems That Regulate Intestinal Epithelial Cell Death Processes and Shedding

**DOI:** 10.3389/fimmu.2017.00841

**Published:** 2017-07-18

**Authors:** Angela M. Patterson, Alastair J. M. Watson

**Affiliations:** ^1^Quadram Institute, Norwich Research Park, Norwich, United Kingdom; ^2^Norwich Medical School, University of East Anglia, Norwich, United Kingdom

**Keywords:** intestinal epithelial cells, shedding, apoptosis, necroptosis, pyroptosis

## Abstract

Intestinal epithelial cells play a fundamental role in maintaining homeostasis. Shedding of intestinal cells in a controlled manner is critical to maintenance of barrier function. Barrier function is maintained during this shedding process by a redistribution of tight junctional proteins to facilitate closure of the gap left by the shedding cell. However, despite the obvious importance of epithelial cell shedding to gut health, a central question is how the extrusion of epithelial cells is achieved, enabling barrier integrity to be maintained in the healthy gut and restored during inflammation remains largely unanswered. Recent studies have provided evidence that excessive epithelial cell shedding and loss of epithelial barrier integrity is triggered by exposure to lipopolysaccharide or tumor necrosis factor alpha. Subsequent studies have provided evidence of the involvement of specific cellular components and signaling mechanisms as well as the functionality of microbiota that can be either detrimental or beneficial for intestinal barrier integrity. This review will focus on the evidence and decipher how the signaling systems through which the mucosal immune system and microbiota can regulate epithelial cell shedding and how these mechanisms interact to preserve the viability of the epithelium.

## Introduction

**Figure 1:**
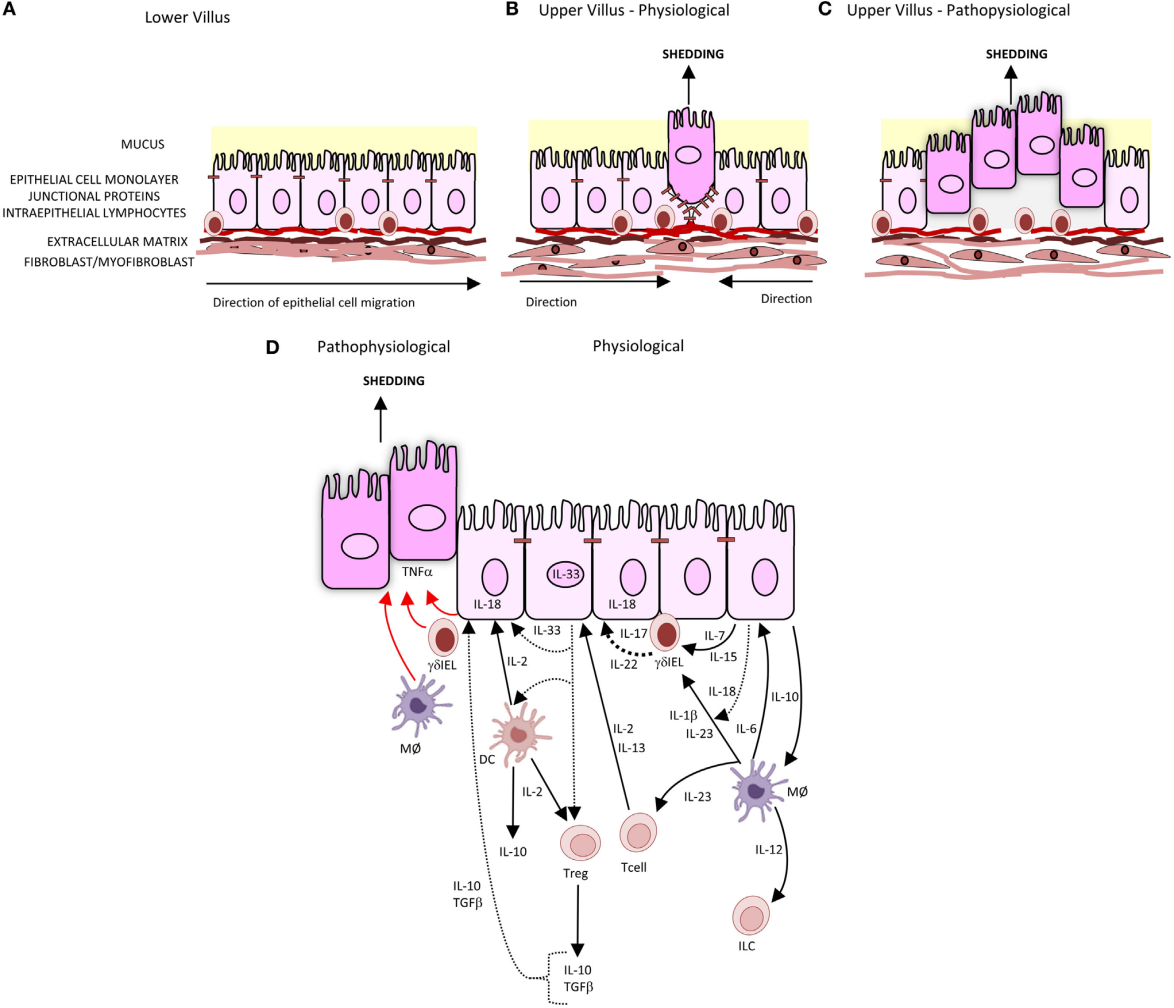
Intestinal epithelial cell shedding. **(A)** In health, epithelial cells migrate up the villus from the base of crypt to the tip. This is achieved through the crawling movement of the epithelial cells through epithelial–substratum interactions between integrins, heparin sulfate proteoglycans, and extracellular matrix. **(B)** At the villus tip, physiological cell shedding with redistribution of tight junctional proteins. **(C)** Pathophysiological cell shedding with multiple cells shed from a single site leading to barrier loss. **(D)** Immunological regulation of cell shedding. Under pathophysiological conditions, TNFα is released by γδIELs, macrophages (MØ), and intestinal epithelial cells (represented by red arrow) resulting in cell shedding. Pathways of cytokines secreted by intestinal epithelial cells, T cells, T regulatory cells (Tregs), and dendritic cells (DC) involved in intestinal epithelial barrier integrity are represented by black arrows, with dashes representing cytokines which have been identified but specific role in barrier integrity and subsequent regulation of cell shedding not yet defined.

The intestinal barrier separates the body from the contents of the intestine. It comprises several elements: a mucus layer containing antibacterial peptides covering the luminal surface of the epithelium; the epithelial cell monolayer, junctional proteins, and intraepithelial lymphocytes (IELs); and a subepithelial layer of extracellular matrix and mesenchymal cells including myofibroblasts and fibroblasts. A central element of this intestinal barrier is the epithelial cell (1). In health, there is a continuous shedding of epithelial cells from villus tip or colonic surface as a result of migration of the epithelial cell up the crypt–villus axis from stem cells at the base of the crypt (Figure 1A). The shedding of epithelial cells is counter-balanced by cell division in the crypt region of the villi to maintain homeostasis and a strict single layer epithelium and integrity of the crypt–villus axis (2–4). In physiological conditions, epithelial cells undergo apoptosis during the shedding process though it remains unclear whether apoptosis initiates the shedding process or is secondary to detachment from the basement membrane (3) (Figure 1B). In contrast to physiological cell shedding, tumor necrosis factor alpha (TNFα)-induced apoptotic cell shedding often results in the shedding of multiple adjacent cells causing a breach in the epithelial monolayer too large to be sealed with subsequent loss of barrier function (5) (Figure 1C).

## Balance of Cell Division, Migration, and Shedding Maintains Barrier Integrity

Epithelial migration is intimately coupled to cell shedding as the two processes must be coordinated to maintain a steady number of epithelial cells on the crypt/villus axis. Until recently, little has been known about the cellular and molecular mechanisms of intestinal epithelial cell migration. This migration is a complex of mechanisms through which each component is intricately balanced. The crawling movement of the epithelial cell along the crypt–villus, axis relies on epithelial cell–substratum interactions regulated by the expression of integrins (6, 7), heparan sulfate proteoglycans (8), growth factor (9), cytokine (10), and chemokine receptors (11) as well as extracellular matrix such as laminins and collagen IV (12).

When the epithelial cell is shed, a discontinuity or gap in the villus epithelial monolayer is created, which could potentially compromise the epithelial barrier. However, in health, normal cell shedding never causes a breach in the epithelial barrier because this gap is plugged by redistribution of tight junction proteins, which include occludin, ZO-1, and the adherens junction protein E-cadherin (13). This redistribution mechanism of tight junction proteins has also been reported in TNFα-induced cells shedding at sites where the gap created by cell shedding has been successfully sealed (14). A further refinement to the extrusion mechanism has been added by the observation that the extrusion of the dying cell is initiated by tension of the dying cell on its neighbors transmitted through cortical contractile actin and a myosin ring at the apex of the dying epithelial cell (15). The redistribution of tight junction proteins results in the modulation of actin filaments, either through actin polymerization with the formation of lamellipodial or actin–myosin interactions forming a ring or a combination of both (16, 17). The mechanics of actin polymerization and lamellipodial formation and actin–myosin interactions are not only dependent on GTPases, Rac1 and Rho (18), respectively, and Cdc42 (19), and trefoil factors (9, 20), but also on many factors including regulation of actin-binding proteins such as villin (21–26), the locality and density of the cell shedding (17, 27), substratum extracellular matrix (28), gap formation (29), and cytokine signaling pathways. Cytokines such as IFNγ and TNFα are involved not only in regulating the remodeling of the junctional proteins (30) but can also be regulated by junctional proteins (31). These cytokines can also act synergistically through the convergence of the β-catenin signaling pathways. IFNγ regulates intestinal epithelial cell proliferation and apoptosis through AKT–β-catenin pathways and Wnt–β-catenin signaling pathways, with TNFα activation of the β-catenin signaling through P13K-AKT and NF-κB signaling (32).

To untangle these complexities, computational modeling of cell division and migration as well as the use of *in vivo* and *in vitro* models using epithelial cell lines and keratinocytes have been used (4, 15, 33–38). The morphological properties of the cells selected for the cellular models are monolayer formation and contractility including the ability to undergo cell division, morphogenesis, and migration to close gap formation caused by injury (39, 40). This has provided an insight into how epithelial cells that line many organs surface operate but how that information can be applied to understand the mechanisms of cell homeostasis and repair within the intestine. Wong and colleagues (33) focused on the migratory positioning and velocity of cells within the crypt and developed a model demonstrating this through the expression and interactions of Eph receptors and ephrins and their regulation cell adhesion. The study highlighted the importance of the cell–cell, cell–substratum, and cytoskeletal organization for maintaining cell migration along the crypt. Parker and colleagues (4) demonstrated how the proliferation of cells within the crypt is the primary force for driving cell migration up the villus and by implication cell shedding. Maintenance of epithelial homeostasis and response to injury is regulated through the expression of signal transduction pathways such as WNT (41, 42) and NOTCH (43, 44) and JAK/STAT pathways and interaction with cytokines. The pathways are highly complex with multiple interactions. For example, JAK3/IL-2/IL2R can result in regulation of villin (45), the STAT5 pathway regulates cellular proliferation of intestinal stem cells (46), and STAT3/IL-22/IL-22R pathways regulate cellular regeneration (47).

The factors determining whether an individual intestinal epithelial cell is shed is not understood. In epithelial cells of the Zebrafish fin, it has been found that the overcrowding and physical stretching of the epithelial cell as it reaches the tip of the fin is sensed by the stretch activated cation selected ion channel Piezo-1. This stimulates extrusion of the epithelial cells through sphingosine 1-phosphate signaling and Rho kinase (37). Furthermore, it has recently been demonstrated that cellular crowding sensed through Piezo1 increases epithelial proliferation in the Zebrafish larvae to preserve overall epithelial homeostasis (38). It is not known whether similar mechanisms occur in the mammalian intestine.

A recent study has suggested that the actin regulatory protein villin might direct the site of intestinal epithelial apoptotic cell shedding on the villus. It regulates cell turnover through the regulation of caspase-3 and caspase-9 apoptotic pathways and regulating actin polymerization and depolymerization (21). Recent data have demonstrated that villin is not only anti-apoptotic but also has pro-apoptotic functions. This function is dependent on the cleavage of villin by proteolytic enzymes. These enzymes, such as meprin, a matrix metalloproteinase, cleaves the villin into fragments, of which the N-terminal villin fragment is pro-apoptotic at the villus tip and can reorganize the actin filaments resulting in cell shedding (48).

## Types of Cell Death Inducing Cell Shedding

A number of types of cell death have been reported intestinal epithelial cells. TNFα-induced apoptotic cell shedding has been studied in some detail. However, it is becoming appreciated that pyroptosis and necroptosis also play a role in intestinal epithelial cell injury (Table 1).

**Table 1:** Intestinal epithelial cell death processes involved in cell shedding (49–56).

Apoptosis	Necroptosis	Pyroptosis	Necrosis
Caspase-3 +ve	Caspase-3 −ve	Caspase-3 −ve	Caspase-3 −ve
Caspase-1 −ve	Caspase-1 +ve	Caspase-1 +ve	Caspase-1 −ve
Tunnel +ve	Tunnel +ve	Caspase-11 (mouse) +ve	Annexin V +ve
Annexin V +ve	RIP3 +ve	Caspase-4 (human) +ve	Propidium iodide +ve
Propidium iodide −ve	RIPK-3 +ve	Caspase-5 (human) +ve	*Tunnel +ve/−ve*
*Caspase-8 +ve/−ve*	Caspase-8 +ve	Gasdermin D +ve	

Apoptosis is mediated through either intrinsic or extrinsic pathways (49, 50). In the intrinsic pathway, cellular injury triggers the release of cytochrome *c* from mitochondria to form an apoptosome in cytosol, comprising cytochrome *c*, apopotic protease factor 1 (APAF-1), and procaspase-9, which triggers activation of a cascade of proteases called caspases which kill the cell. In the extrinsic pathway, apoptosis is triggered by the binding of external proteins such as TNFα or FasL to their cognate receptors expressed on the surface of the target cell. The binding of the ligand to the receptor stimulates the activation of caspase-8 through a series of intermediate proteins to cause apoptosis (51). In a mouse model of rapid small intestinal epithelial cell shedding and apoptosis developed by Watson and colleagues (5, 14, 16, 52), it has been demonstrated that TNFα release in the lamina propria caused cell shedding *via* the TNF receptor 1. The TNFα then activates NF-κB pathway. A differential sensitivity of cell shedding to NF-κB pathways was observed with NF-κB1 decreasing sensitivity, while NF-κB2 increases the sensitivity of epithelial cell shedding to lipopolysaccharide (LPS). Studies of the mechanism of cell shedding have shown that activation of caspase-3 by TNFα cleaves and activates Rho-associated protein kinase (ROCK1) and the phosphorylation of myosin light chains resulting in the membrane blebbing formation in apoptotic cells. Inhibition of either of these enzyme activities arrests cell shedding after its initiation such that the shedding process is incomplete (14, 15). In addition, it has been reported that synthesis of sphingosine-1-phosphate by dying cell binds to the S1P(2) receptor in neighboring cells to activate myosin contraction to extrude the dying cell out of the epithelial monolayer (53).

Ubiquitin-dependent signaling activated by pattern recognition receptors (PRRs) mediates activation of NF-κB transcription factors as well as the MAP kinases p38 and JNK. NF-κB1 and MAPK expression reduces cell shedding, while NF-κB2 increases shedding. NF-κB is required for expression of downstream cytokines and chemokines such as TNFα, IL-6, IL-1β, and CCL20. Data to date demonstrate an action of PRRs in intestinal inflammation and epithelial apoptosis; therefore, it is plausible that aspects of the innate immune system may regulate cell shedding.

The mode of cell death is dependent on the activation of various cellular signaling pathways after initial cytokine stimulation. The differences have been highlighted recently by the groups of Günther et al. and Rauch et al. (52, 54). In the absence or inactivation of caspase-8, TNFα induces necroptosis at the base of the crypt with loss of Paneth cells *via* RIP-3 kinase. This is relevant to Crohn’s disease as necroptosis occurs in the intestinal crypt (55). Caspase-8 acts as a type of switch. When functional, it initiates apoptosis which is a benign form of cell death from the point of view of the whole animal. However, when caspase-8 is not functional, cell death still occurs but *via* RIP3-kinase-dependent necroptosis which affects multiple cell types in a number of organs with increased mortality (52). Rauch and colleagues demonstrated the induction of apoptosis through caspase-8 activation and interaction with inflammasomes. Inflammasomes in inflammation regulate cell death through the activation of caspase-1 resulting in the expulsion of cells or pyroptosis. This mode of action can be induced through microbial ligands binding to NAIP family members of the inflammasome complex (56).

## Bacterial Entry and Epithelial Cell Shedding

When shedding of multiple adjacent apoptotic cells creates gaps that are too large to be plugged by the redistribution of apical junctional proteins, as frequently occurs when TNF concentrations are high, the epithelial barrier is breached at the shedding site (14). In clinical studies using confocal endomicroscopy, this has been shown to trigger relapse of inflammatory bowel disease (14). This allows the entry of bacteria such as *Listeria* (57), antigens, and toxins from the lumen, which act to amplify inflammatory reactions within the lamina propria. However, apoptotic cell shedding can be an important mechanism to expel epithelial cells invaded by pathogenic bacteria and thereby reducing the chance of bacterial colonization as well as localizing inflammatory reactions. To this end, pathogenic bacteria, such as *Shigella, Citrobacter*, and *Salmonella*, have evolved to prevent cell shedding through the production of bacterial effector proteins. One effector protein secreted by these bacteria is the protein OspE that enhances epithelial cell–matrix interactions through binding of the integrin-linked kinase of the epithelial cell to the cells actin cytoskeleton resulting in increased integrin expression and thereby increased focal adhesions to the extracellular matrix (58, 59). This evasive mechanism results in bacterial colonization and inflammatory reactions within the intestine. However, this bacterial evasive mechanism relies on an interaction between the epithelial cell and underlying matrix *via* the integrin-linked kinase, which can only take place in the crypt and lower villus (58, 59). Although *Salmonella* can inhibit cell shedding, and thereby interfere with the epithelial cell response to bacterial infection, it is not the only mechanism of defense by the epithelial cells. This mechanism is through the formation of inflammasomes complexes, caspase-1 activation, and the production of cytokines and ultimately pyroptotic cell death (60), although recent work has demonstrated that this mechanism can result in apoptotic and pyroptotic cell death *via* caspase-8 activation (52, 61).

## Regulation of Cell Shedding by the Mucosal Immune System

Intraepithelial lymphocytes within the epithelial monolayer have normally been associated with celiac disease; however, recent date indicate that they may have a central role in epithelial barrier function. Interestingly, recent data from Edelblum and colleagues (62) have demonstrated that γδ-IELs can migrate along the epithelium by an occludin-dependent mechanism. Given that occludin is redistributed to surround the shedding cell during expulsion, it is an attractive hypothesis that the IELs might participate in the regulation of cell shedding through occludin-dependent mechanisms. IELs could initiate epithelial cell restitution by stimulating epithelial cell migration into the gap created by cell shedding. They might also signal to the epithelial cells adjacent to the shedding cells to stimulate cytoskeletal reorganization. Migration of IELs within the epithelium can also be regulated by the chemokine–chemokine receptor interaction such as CCL25–CCR9 (63) as well as through the expression of chemokine receptors CCR5, CX3CR1, and CCR3 (64). Chemokine regulated migration of IELs could potentially direct IELs to sites of cell shedding. IELs could potentially also regulate the responses of other cell populations, such as subepithelial myofibroblasts and macrophages. Such subepithelial responses may be important in the prevention of paracellular migration of opportunistic pathogenic (65, 66) and commensal bacteria (67).

Both innate and adaptive immunity are hypothesized to regulate or respond to cell shedding. Within the innate immune system that comprises monocytes/macrophages, dendritic cells, innate lymphoid cells, and epithelial cells, microbes are recognized by PRRs such as toll-like receptors and nucleotide oligomerization domains (NODs) expressed on these cells. We have found that *Bifidobacterium breve* significantly reduce LPS and TNFα-induced epithelial cell shedding through a NOD2-dependent mechanism that requires the exopolysaccharide of the Bifidobacteria (68).

Although there that been innumerable studies of components of the adaptive and innate immune systems regulating mucosal damage, only few studies that specifically investigated the regulation of epithelial cell shedding (Figure 1D). Mechanistic studies have demonstrated a role for T regulatory cells in both adaptive and innate immunity. Production of cytokines IL-10, IL-4, and IL-13 is critical for suppression of pro-inflammatory cytokine responses from other immune cells such as monocytes/macrophage and thus could reduce TNFα-induced cell shedding. IL-13 also downregulates the effects of LPS-induced endotoxin. Its effects of LPS-induced cell shedding have not been reported. IL-13 has been shown to modulate intestinal epithelial tight junctions, claudin-2, and apoptosis and therefore potentially cell shedding (69). The cytokines, such as IL-10, IL-21, IL-22, IL-23, and IL-6, activate STAT3 and, in addition to IL-13, are also regulated through STAT3. Inhibition of STAT3 blocks the anti-apoptotic activity of IL-6 (70); therefore, it is possible that inhibition of STAT3 may also disrupt the immunosuppressive action of IL-13 and IL-10, which in turn modulates TNFα production and thereby epithelial shedding and apoptosis.

## Conclusion

Important advances have been made in our understanding of the maintenance of epithelial integrity in health and disease. The mechanisms of extrusion of epithelial cells are now being unraveled though it remains unclear what the determinants are of an individual epithelial cell being shed. A number of studies of cytokines and chemokines have demonstrated their importance in epithelial integrity they have not specifically addressed their role in the regulation of cell shedding itself. It is now appreciated that a number of types of cell death can trigger epithelial extrusion with increasing examples of necroptosis and pyroptosis being reported in addition to apoptosis. There is now also an increasing understanding that epithelial cell shedding can be a protective mechanism against infection through expulsion of invading pathogens. Further studies are likely to reveal therapeutic targets for inflammatory and infective bowel disease.

## Author Contributions

AP collected and analyzed data, drafted the manuscript, and contributed toward and approved the final manuscript. AW drafted the manuscript and contributed toward and approved the final manuscript.

## Conflict of Interest Statement

The authors declare that the research was conducted in the absence of any commercial or financial relationships that could be construed as a potential conflict of interest. The reviewer, SK, and handling Editor declared their shared affiliation, and the handling Editor states that the process nevertheless met the standards of a fair and objective review.
